# Influence of seismic strain stress on evolution law of microcracks in concrete TPB tests using AE technology

**DOI:** 10.1038/s41598-026-49968-0

**Published:** 2026-04-22

**Authors:** Dong Xiao, Lina Wen, Yong Cao, Shuang Chen, Yinling Dou, Li Tang

**Affiliations:** 1 Sichuan Provincial Engineering Research Center of Rail Transit Lines Smart Operation and Maintenance, Chengdu, 610218 China; 2 Sichuan Highway Planning, Design and Research Institute Ltd, Survey, Chengdu, 610041 China; 3 Chengdu Productivity Promotion Center, Chengdu, 610041 China

**Keywords:** Concrete, Seismic strain rate, TPB, AE technology, Fracture, Cracks evolution, Engineering, Materials science, Natural hazards, Solid Earth sciences

## Abstract

Since the fracture properties and failure modes of concrete are deeply rate-dependent, changes in crack resistance and failure mechanism of concrete, under seismic strain rates, can provide critical insights for structural safety withstanding seismic load. In this study, the dynamic fracture characteristics and micro-mechanisms of concrete under a wide of strain rates range from the static to the seismic (10^− 6^s^− 1^~ 10^−2^s^− 1^) were explored using three-point bending (TPB) tests combined with Acoustic Emission (AE) monitoring. The results reveal a significant positive correlation between strain rate and mechanical performance. As the strain rate increases, the peak load increases by up to 37.1%, and the fracture energy rises by up to 36.7%, demonstrating a distinct pseudo-strengthening effect. Microscopically, the failure mechanism transitions from ductile interfacial cracking, where cracks deflect along the interface transition zone (ITZ), to brittle transgranular cracking, where aggregates are fractured directly. AE analysis further indicates a shift in the dominant fracture mode from Mode I (tensile) to Mode II (shear), with the proportion of shear cracks increasing from 16.2% to 53.8%. In addition, the spatial distribution of AE events becomes highly concentrated near the pre-crack tip, signifying a transition to brittle failure. These findings provide critical insights into the dynamic fracture mechanisms of quasi-brittle materials, highlighting the inherent trade-off between fracture strength enhancement and ductility reduction under rapid loading conditions, which is essential for seismic engineering and structural safety assessments.

## Introduction

**Fig. 1:**
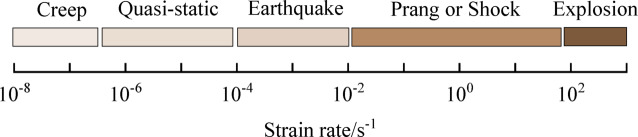
Classification of strain rates for different types of loads^2–4^.

As the most primary structural material in civil engineering, the dynamic mechanical behavior of concrete under seismic strain rate is closely related to the seismic safety and disaster prevention and control of structures^1^. Bischoff and Perry classified various types of loads according to the level of strain rate^2–4^, as illustrated in Fig. 1. The seismic strain rate usually ranges from 10^− 4^ to 10^− 2^ s^− 1^. The influence of earthquake-level strain rates on the mechanical behavior of concrete is different not only from that of impact or blast loads, but also essentially distinct from that under quasi-static loading conditions. A large number of studies have shown that the strength, deformation capacity and fracture mode of concrete all exhibit significant strain rate sensitivity, that is, with the increase of strain rate, the dynamic compressive and tensile strengths are significantly improved, while the failure mode changes from ductile to brittle^3,5–7^.

The research on the dynamic properties of concrete can be traced back to the work in 1917^8^. The consensus view is that as the strain rate increases, the uniaxial compressive strength, initial elastic modulus, tangent modulus at the peak stress, and energy absorption capacity of concrete all increase, the slope of the descending section becomes steeper, the Poisson’s ratio shows no significant change, and the shape of the stress-strain curve shows no significant difference^2,9–11^. However, the variation laws of the critical strain and ultimate strain at the peak stress remain undetermined. Based on summarizing the majority of experimental results, the European Concrete Association (CEB) stipulated a quasi-static strain rate and recommended the relative dynamic increase coefficients of the compressive strength, peak strain, and elastic modulus of concrete materials under different dynamic strain rates compared to the quasi-static strain rate^12^.

At present, Scholars widely employ dynamic increase factor and the fracture work method in the research on the dynamic fracture characteristics of concrete^13–16^. For instance, Wu^17^when studying the strain rate effect on the fracture behavior of concrete, conducted a detailed investigation on the influence of different strain rates on stress-strain curves, peak stress, elastic modulus, Poisson’s ratio and dynamic amplification factor. Zhu et al^18^. conducted TPB at different loading rates, established a bilinear softening constitutive model using the finite element method and modified fracture energy, and applied it to predict the fracture behavior of concrete.

However, most existing studies have focused on high strain rate conditions such as impacts or explosions. There is still a lack of in-depth and systematic understanding of the dynamic fracture evolution mechanism of concrete during the micro-crack initiation and propagation to macroscopic connection within the seismic strain rate range, especially the spatiotemporal characteristics of damage accumulation. The formation and propagation of cracks is a complex and progressive process, and it is also a key mechanism leading to the fracture failure of concrete. During the crack propagation process, its morphology and state can reflect the crack-resistance ability and cracking mechanism of concrete. Traditional mechanical testing methods can often only record macroscopic responses such as load - displacement curves, and it is difficult to capture the nucleation and propagation process of microcracks inside the material in real time, which leads to difficulties in explaining the internal mechanism of the strain rate effect. AE technology, as a non-destructive testing method, can sensitively capture the elastic wave signals released due to the generation and propagation of microcracks during the deformation of materials, providing a unique perspective for revealing the internal damage evolution of concrete^19–26^. By analyzing the variation laws of characteristic parameters of AE events such as count rate, energy distribution and b - value with the strain rate, it is expected to establish the intrinsic correlation between macroscopic mechanical response and microscopic damage mechanism, and clarify the differences in the selection of fracture crack paths and their energy dissipation mechanisms under different strain rates.

To this end, this study focuses on the strain rate range corresponding to earthquake levels, conducting TPB tests on pre-notched concrete beams and AE monitoring. Fracture mechanics data and AE signals are synchronously collected. The focus is on analyzing the influence laws of different strain rates on the peak stress, fracture toughness, fracture energy and failure mode of concrete. Combined with AE characteristic parameters, the location distribution of AE sources and the identification of microcrack types, the strain rate effect, evolution process and mechanism of concrete fracture damage are deeply analyzed. This study aims to provide the first comprehensive analysis of fracture behavior and AE response mechanisms within the seismic strain rate range, bridging a critical gap between quasi-static and high-rate dynamic regimes and can also provide important theoretical basis and technical support for the seismic design, damage assessment and toughness improvement of concrete structures in high-intensity areas.

## Experimental program

### Test materials and mix proportions

The cement used was P·O 42.5 ordinary Portland cement produced by Leshan Fuxiang Building Materials Co., Ltd., with quality and performance meeting industry standards. Fly ash was purchased from Chengdu Shenghong Building Materials Co., Ltd., classified as Grade II, with a loss-on-ignition of less than 5%. Fine aggregate consisted of natural medium-coarse river sand with a fineness modulus of 2.60 and particle size ranging from 0.15 to 4.75 mm. Coarse aggregate was continuously graded crushed stone with a particle size of 5 ~ 20 mm and a specific gravity of 2.56. A polycarboxylate-based high-efficiency water reducer was used, providing a water reduction rate of 30%. Mixing water was municipal tap water. According to the “Code for Design of Ordinary Concrete Mix Proportions” (JGJ55-2011), C30 concrete was designed. The specific mix proportions and the 28-day cube compressive strength of the concrete are presented in Table 1.

**Table 1:** Mix proportion for concrete for TPB tests.

Cement	Water	Sand	Crushed stone	Fly ash	Compressive strength/MPa
312	178	1056	1857	136	31.5

### Specimen preparation

TPB specimens were prepared according to the mix proportions listed in Table 1. The specimen dimensions were 400 mm × 100 mm × 100 mm, with three specimens per group and a total of 15 specimens across five groups. The specimens were cured under standard conditions for 28 days. A pre-crack was manufactured at the mid-span cross-section of each specimen, with an initial crack depth ratio of 0.4. A T-shaped steel plate (40 mm high and 3.5 mm thick) was embedded during casting to create the pre-crack. Specific specimen dimensions are shown in Fig. 1.

**Fig. 2:**
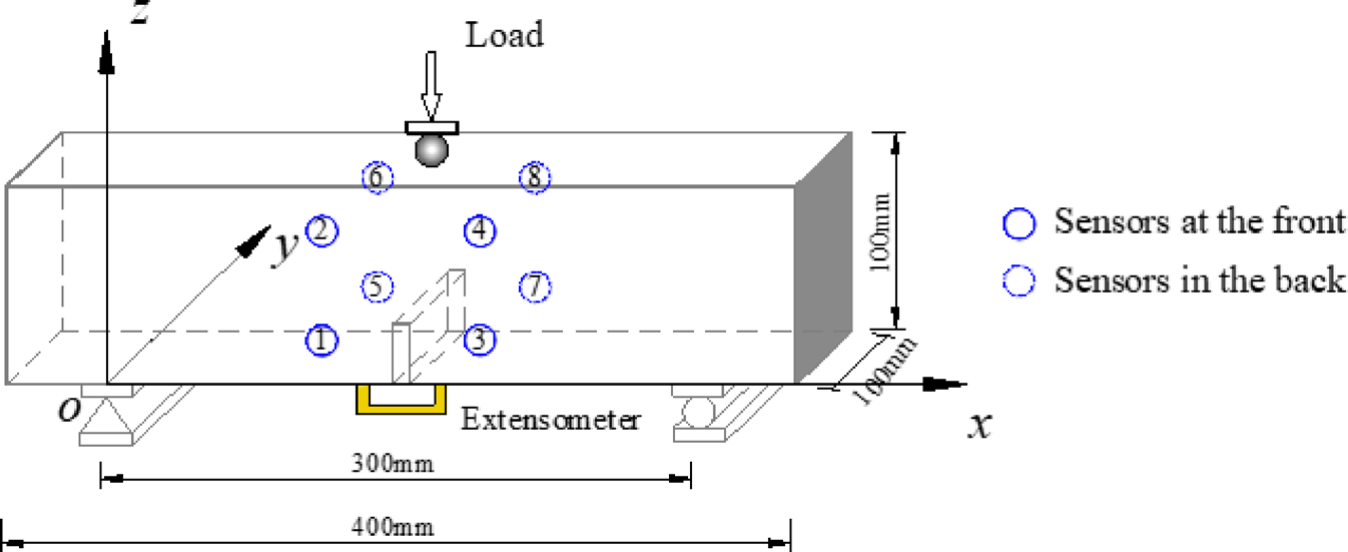
The schematic diagram of test device.

### TPB test scheme

The testing equipment used was an MTS-322 series microcomputer-controlled electro-hydraulic servo universal testing machine, employing displacement-controlled loading. The loading control method was displacement control, with five levels of strain rates (defined as the ratio of loading displacement rate to specimen height): $\dot {\varepsilon }$=10^− 6^ s^− 1^, 10^− 5^ s^− 1^, 10^− 4^ s^− 1^, 10^− 3^ s^− 1^, and 10^− 2^ s^− 1^, covering a range from quasi-static to earthquake-level strain rates^2–4,27^. The span *S* is 300 mm. The crack mouth opening displacement (CMOD) of the pre-crack was measured using a clip-on extensometer, while the mid-span deflection (*δ*) was recorded by a displacement transducer. The applied load (*P*) during the test was automatically collected by the universal testing machine. The extensometer and displacement transducer were connected to a dynamic data acquisition system for real-time recording. A schematic diagram of the TPB test setup is shown in Fig. 2.

### AE monitoring

AE technology was used to monitor the fracture process of the concrete. The AE equipment employed was the SAMOSTM AE system manufactured by Physical Acoustics Corporation. A total of 8 sensors were used for signal acquisition, with 4 sensors placed on each side (front and back) of the specimen to locate AE sources (Fig. 2); the sensor coordinates are detailed in Table 2. The sensors were fixed at predetermined positions on the specimen surface using adhesive tape, and petroleum jelly was used as a coupling agent to ensure close contact between the sensors and the concrete surface. The type of the AE sensor was PK6I, with operating parameters as follows: resonant frequency ≤ 55 kHz, signal acquisition threshold of 40 dB, sampling rate of 3 MHz, and amplifier gain of 35 dB. Prior to testing, the AE wave velocity was determined through the pencil lead break (PLB) to determine the time parameters of the concrete. 2.5 mm lead cores were taken from the lead-breaking points of three groups of concrete specimens. The lead cores were placed at a 30° angle to the surface of the specimens and the lead-breaking process was carried out once, followed by five consecutive lead-breaking tests. The average wave velocity of the longitudinal wave in our concrete material was calculated to be 3850 ± 150 m/s.

**Table 2:** Co-ordinates of the AE sensors.

Sensor number	x/mm	y/mm	z/mm
1	100	0	30
2	100	0	70
3	200	0	30
4	200	0	70
5	100	100	30
6	100	100	70
7	200	100	30
8	200	100	70

### Determination of fracture parameters

Through the TPB test, the load-crack mouth opening displacement curve (P-CMOD curve), the load-mid-span deflection curve (*P*-*δ*), as well as the crack initiation load and ultimate load (*P*_ini_、*P*_max_) can be obtained. The concrete fracture model employs the double-*K* fracture model, which includes two important parameters: the initiation toughness *K*ini Ic and the unstable toughness *K*un Ic. When the specimen’s toughness *K* < *K*ini Ic, the crack has not initiated. When *K* = *K*un Ic, the crack begins to propagate stably from the tip of the pre-made crack. When *K*ini Ic < K < *K*un Ic, the crack is in a state of stable propagation. When *K* = *K*un Ic, the crack is in a critical state and begins to propagate unstably. When *K* > *K*un Ic, the crack is in a state of unstable propagation^28^. The calculation formulas for *K*ini Ic, *K*un Ic, and the fracture energy (*G*_F_) are as follows^29^:

1 $$K_{{{\mathrm{Ic}}}}^{{{\mathrm{ini}}}}=\frac{{1.5{P_{{\mathrm{ini}}}}S}}{{{h^2}b}}\sqrt {{a_0}} f\left( \alpha \right)$$

2 $$f\left( \alpha \right)=\frac{{1.99 - \frac{{{a_0}}}{h}\left( {1 - \frac{{{a_0}}}{h}} \right)\left( {2.15 - 3.93\frac{{{a_0}}}{h}+2.7{{\left( {\frac{{{a_0}}}{h}} \right)}^2}} \right)}}{{\left( {1+2\frac{{{a_0}}}{h}} \right){{\left( {1 - \frac{{{a_0}}}{h}} \right)}^{3/2}}}}$$

3 $$K_{{{\mathrm{Ic}}}}^{{{\mathrm{un}}}}=\frac{{1.5{P_{{\mathrm{max}}}}S}}{{{h^2}b}}\sqrt {{a_{\mathrm{c}}}} f\left( \alpha \right)$$

4 $$f\left( \alpha \right)=\frac{{1.99 - \frac{{{a_{\mathrm{c}}}}}{h}\left( {1 - \alpha } \right)\left( {2.15 - 3.93\frac{{{a_{\mathrm{c}}}}}{h}+2.7{{\left( {\frac{{{a_{\mathrm{c}}}}}{h}} \right)}^2}} \right)}}{{\left( {1+2\frac{{{a_{\mathrm{c}}}}}{h}} \right){{\left( {1 - \frac{{{a_{\mathrm{c}}}}}{h}} \right)}^{3/2}}}}$$

5 $${a_{\mathrm{c}}}=\frac{2}{\pi }\left( {h+{h_0}} \right)\arctan \sqrt {\frac{{Eb{\mathrm{CMO}}{{\mathrm{D}}_{\mathrm{c}}}}}{{32.6{P_{{\mathrm{max}}}}}} - 0.1135} - {h_0}$$

6 $$E=\frac{1}{{{c_{\mathrm{i}}}b}}\left( {3.7+32.6{{\tan }^2}\left( {\frac{2}{\pi }\frac{{{a_0}+{h_0}}}{{h+{h_0}}}} \right)} \right)$$

7 $${G_{\mathrm{F}}}=\frac{{\int_{0}^{\infty } {P\left( \delta \right){\mathrm{d}}\left( \delta \right)+mg{\delta _{\hbox{max} }}} }}{{b\left( {h - {a_0}} \right)}}=\frac{{{W_0}+{W_1}}}{{{A_{{\mathrm{lig}}}}}}$$

Where, *K*ini Ic and *K*un Ic refer to the initiation toughness and unstable toughness, respectively (MPa·mm^1/2^); *P*_ini_ and *P*_max_ denote the crack initiation load and peak load, respectively (kN); *S*, *h* and *b* are the span, height, and thickness of the specimen, respectively (mm); *a*_0_ and *a*_c_ represents the pre-manufactured crack length and the critical equivalent crack length, respectively (mm); *h*_0_ is the thickness of the fixed clip gauge knife-edges; *D*_c_, i.e., CMOD_c_, refers to the critical crack mouth opening displacement (mm); E represents the calculated elastic modulus of concrete (GPa); *c*_i_ is the initial compliance, *c*_i_ = CMOD_i_/*P*_i_; *G*_F_ refers to the fracture energy of the concrete specimen (N·m^− 1^); *W*_0_ is the work done by the external force on the concrete specimen (J); *W*_1_ is the work done by the self-weight of the concrete specimen (J); *A*_lig_ represents the fracture zone area of the concrete specimen (mm^2^); *m* is the mass of the concrete specimen over the span (kg); *g* denotes the acceleration due to gravity, taken as 9.8 m/s²; *δ*_max_ is the deflection at the final failure point of the concrete specimen (mm).

## Results and analysis

### Fracture surface morphology

The fracture surface morphology can intuitively reflect the final crack propagation behavior during concrete fracture failure. The concrete exhibits two typical failure modes: interfacial cracking (crack deflection) and transgranular cracking (crack penetration)^30^. Interfacial cracking refers to the behavior where a crack propagating in the mortar matrix reaches the edge of an aggregate particle but cannot propagate through or penetrate it, thus continuing its path along the ITZ, resulting in the formation of an interfacial crack. Transgranular cracking refers to the behavior where the crack in the mortar matrix extends to the edge of the aggregate particle, causing the aggregate itself to fracture and be penetrated by the crack, thereby forming an aggregate crack.

**Fig. 3:**
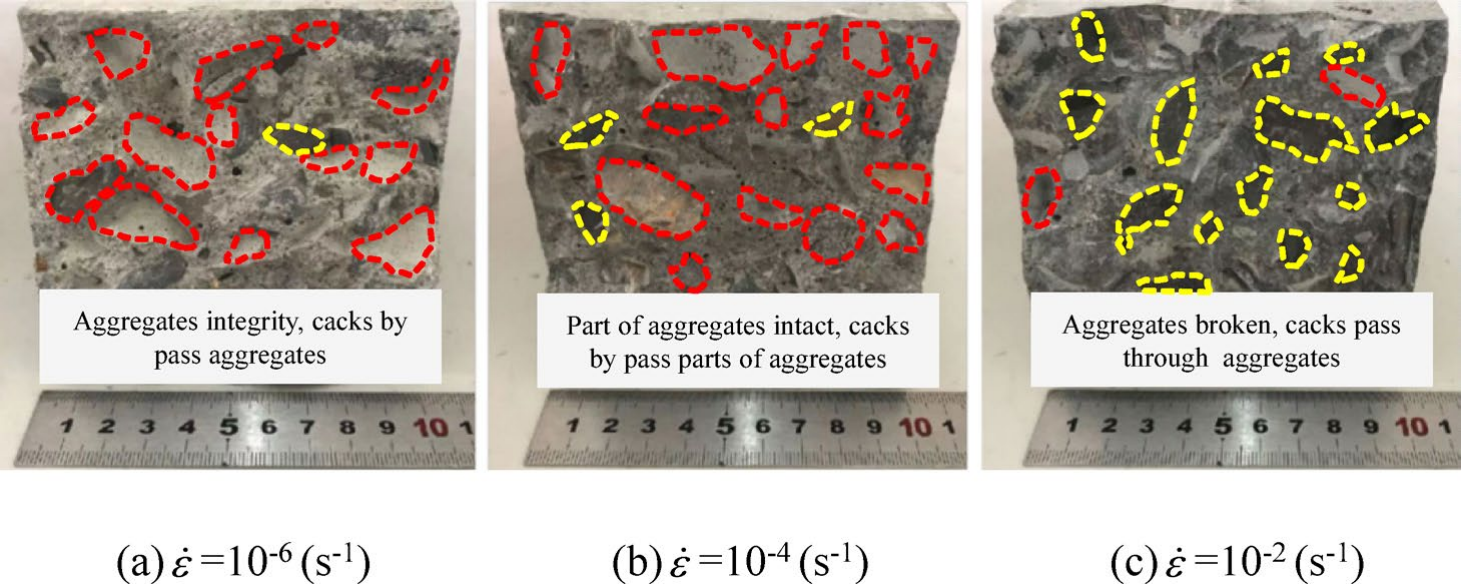
Cross-sectional view of the fracture and destruction of some concrete specimens at different strain rates.

Figure 3 shows fracture surface images of concrete under different strain rates, where red circles indicate exposed ITZs and yellow circles represent fractured coarse aggregates. As shown in the figure, at lower strain rates, the fracture surface is relatively rough and uneven, with the crack path circumventing coarse aggregates and primarily propagating along the ITZs, resulting in a high exposure rate of coarse aggregates, which is characteristic of “interfacial cracking.” With increasing strain rate, the fracture surface becomes smoother, exhibiting more fractured coarse aggregates and a darker fracture surface color, indicating “transgranular cracking.” This occurs because, at low strain rates, cracks have sufficient time for stress redistribution and thus preferentially propagate along the weakest paths—namely, the ITZ—minimizing energy dissipation. This results in numerous intact, exposed coarse aggregates, which is characteristic of “interface-controlled” failure. As the strain rate increases, stress concentrations develop at the crack tip, leaving insufficient time for optimal path selection. Crack propagation is then governed by the instantaneous stress field distribution. Due to the increased energy input rate, the crack path is forced to penetrate more coarse aggregates to dissipate large amounts of energy, leading to an “aggregate-controlled” failure mode.

### P-CMOD curve

**Fig. 4:**
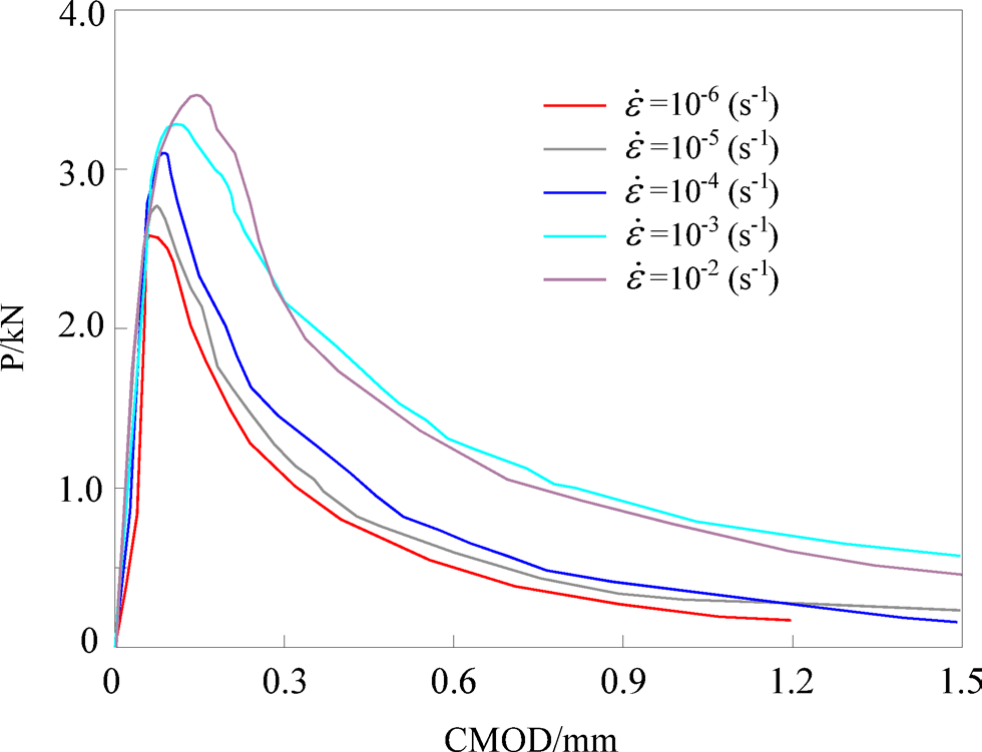
P-CMOD curves at different strain rates.

The load-crack mouth opening displacement (P-CMOD) curve can reflect the relationship between the vertical load and the crack mouth opening displacement during the loading process of a specimen. Figure 4 shows the P-CMOD curves under different strain rates. The P-CMOD curves of different specimens exhibit similar trends, showing a three-stage characteristic: (1) Crack non-propagation stage, i.e., from the beginning of loading until cracking occurs. In this stage, the curve presents a linear relationship; (2) Stable crack propagation stage, where the P-CMOD curve transitions from linear to nonlinear, and the rate of increase in crack mouth opening displacement with increasing load is higher than that in the linear stage. The fracture process zone forms during this stage; (3) Unstable crack propagation stage, i.e., the descending segment of the P-CMOD curve after the peak load, during which the load gradually decreases as the crack mouth opening displacement increases, until the specimen fails by complete penetration of the crack.

It can also be observed that the higher the strain rate, the greater the peak load *P*_max_. When the strain rate increases from 10^− 6^ s^− 1^ to 10^− 5^ s^− 1^, 10^− 4^ s^− 1^, 10^− 3^ s^− 1^, and 10^− 2^ s^− 1^, *P*_max_ increases by 10.4%, 20.5%, 30.1%, and 37.1%, respectively. The reason for this pseudo-strengthening effect is that concrete is a porous material, storing large amounts of water within its internal microcracks, capillaries, and aggregate-mortar interfaces^31^. As the strain rate increases, the water cannot be expelled in time, resulting in enhanced viscous stresses. This is equivalent to adding an additional “damper” at the microscale that impedes crack propagation^32^. To overcome this extra viscous resistance and reach the critical state of unstable crack propagation (i.e., the peak point on the curve), a higher external load must be applied, thereby increasing the peak load.

### Dual K fracture parameter and fracture energy

According to the dual K fracture model proposed by Xu and Reinhardt^33^, the initiation toughness *K*ini Ic and the unstable toughness *K*un Ic are the most important parameters among the dual K fracture parameters. *K*ini Ic refers to the critical stress intensity factor when a macroscopic crack begins to stably propagate from the crack tip, marking the transition point from the crack non-propagation stage to the stable crack propagation stage. *K*un Ic represents the magnitude of the stress field near the crack tip under critical unstable fracture conditions, indicating the transition point from the stable crack propagation stage to the unstable crack propagation stage. Fracture energy *G*_F_ is one of the key parameters characterizing material fracture performance, representing the energy consumed per unit area during crack propagation in concrete, i.e., the energy dissipation capacity.

**Fig. 5:**
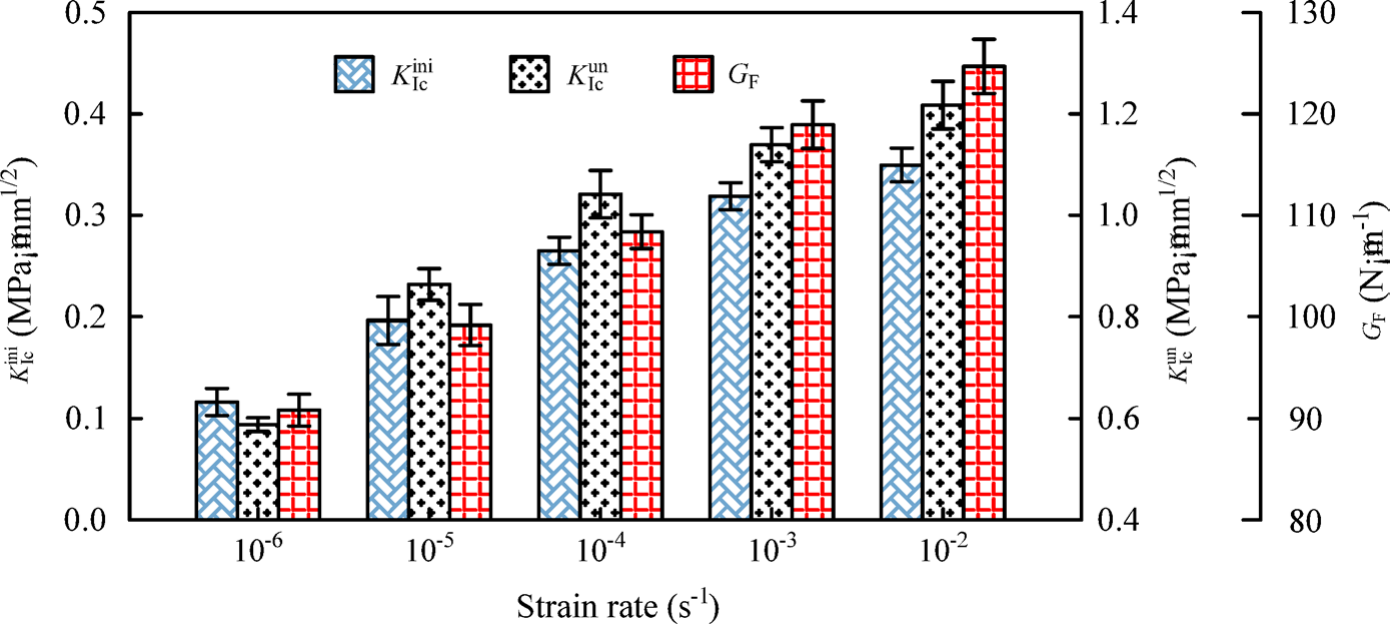
The influence of different strain rates on the fracture parameters of concrete.

Combining the P-CMOD curve, the dual *K* fracture parameters and fracture energy of the specimens were calculated using Eq. (1)~(7), with results shown in Tables 3 and 4. To more intuitively reflect the influence of strain rate on fracture parameters, data for *K*ini Ic, *K*un Ic, and *G*_F_ were plotted as bar charts, as shown in Fig. 5. It can be observed that the dual *K* fracture parameters exhibit a clear correlation with strain rate. At strain rates of 10^− 6^ s^− 1^ to 10^− 5^ s^− 1^, 10^− 4^ s^− 1^, 10^− 3^ s^− 1^, and 10^− 2^ s^− 1^, the corresponding *K*ini Ic values are 0.12, 0.20, 0.27, 0.32, and 0.35 MPa·m^1/2^, respectively, while the corresponding *K*un Ic values are 0.60, 0.88, 1.04, 1.15, and 1.23 MPa·m^1/2^, respectively. Regarding the critical crack mouth opening displacement (CMOD_c_) in the Table 3, a distinct trend is observed: it decreases from 0.152 mm to 0.107 mm and further to 0.086 mm as the strain rate increases from 10^− 6^ s^− 1^ to 10^− 4^ s^− 1^, indicating a reduction in ductility. However, at higher strain rates (10^− 3^ s^− 1^ and 10^− 2^ s^− 1^), CMOD_c_ increases to 0.709 mm and 0.664 mm, respectively. This phenomenon is attributed to the significant strain rate effect on the peak load (*P*_max_). As shown in Table 3, *P*_max_ increases steadily with the strain rate. The substantial enhancement in bearing capacity at high strain rates leads to a higher energy storage in the specimen, resulting in a larger critical displacement at the point of instability despite the material exhibiting more brittle characteristics.

Meanwhile, *G*_F_ also exhibits a significant strain rate effect. When the strain rate increases from 10^− 6^ s^− 1^ to 10^− 5^ s^− 1^, 10^− 4^ s^− 1^, 10^− 3^ s^− 1^, and 10^− 2^ s^− 1^, *G*_F_ increases by 9.5%, 19.1%, 29.4%, and 36.7%, respectively. The underlying reasons are as follows: with increasing strain rate, the viscous effects near the crack tip become more pronounced^9^, leading to higher threshold stresses required to overcome this viscous resistance, thereby increasing the initiation toughness; high strain rates induce non-uniform stress distribution^11–13^, resulting in concentrated fracture processes, straighter crack paths, and extensive shearing of high-strength coarse aggregates, which consume substantial energy and increase the unstable toughness. Since the unstable toughness reflects the overall fracture energy of the material^34–37^, the fracture energy also increases accordingly.

**Table 3:** Calculation results of concrete double K fracture parameters at different strain rates.

Strain rate (s^− 1^)	*P*_ini_ (kN)	*P*_max_ (kN)	CMOD_c_ (mm)	E (GPa)	Kini Ic (MPa·m^1/2^)	Kun Ic (MPa·m^1/2^)
10^− 6^	2.06	2.59	0.152	22.4	0.12	0.60
10^− 5^	2.44	2.86	0.107	24.7	0.20	0.88
10^− 4^	2.71	3.12	0.086	25.9	0.27	1.04
10^− 3^	2.93	3.37	0.709	28.2	0.32	1.15
10^− 2^	3.07	3.55	0.664	31.2	0.35	1.23

**Table 4:** Calculation results of concrete fracture energy at different strain rates.

Strain rate (s^− 1^)	W_0_ (J)	W_1_ (J)	A_lig_ (mm^2^)	G_F_ (*N*·m^− 1^)
10^− 6^	0.39	0.16	6000	91.7
10^− 5^	0.43	0.18	6000	100.4
10^− 4^	0.46	0.19	6000	109.2
10^− 3^	0.51	0.21	6000	118.7
10^− 2^	0.53	0.22	6000	125.3

### AE characteristic parameters

**Fig. 6:**
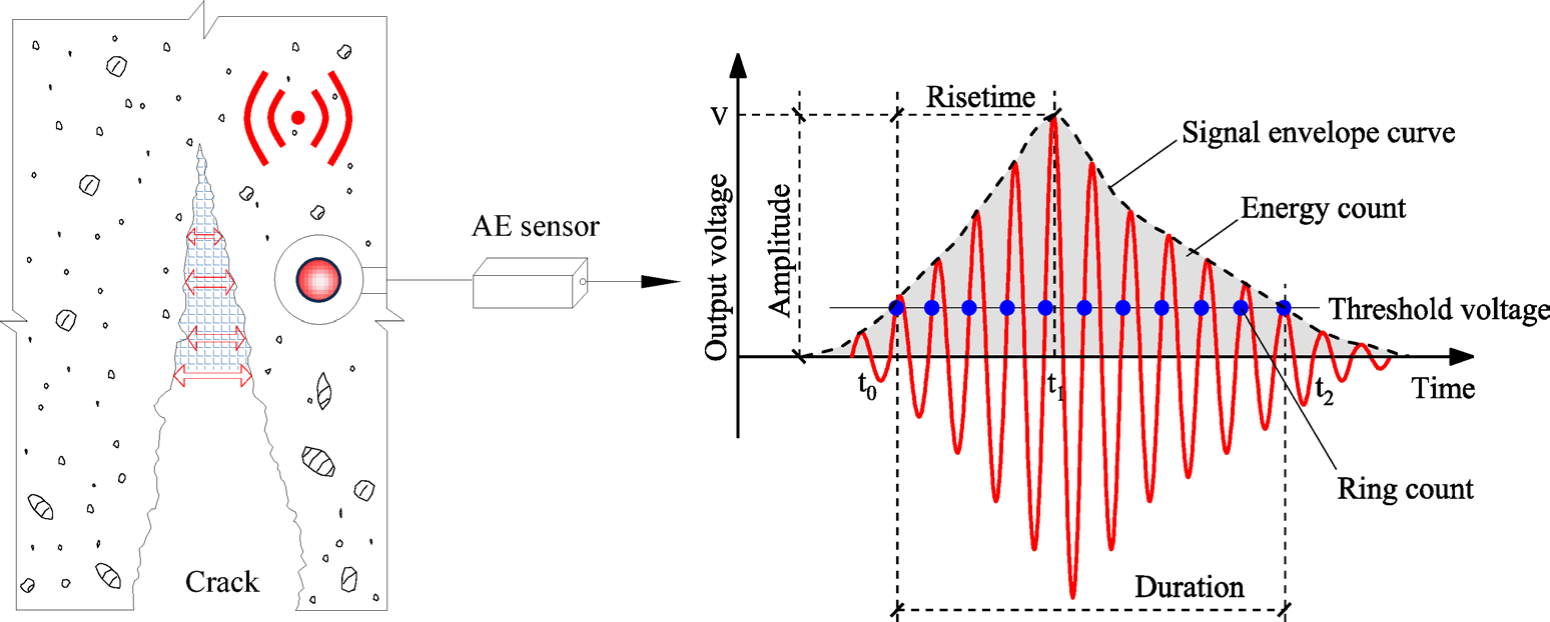
Meaning of AE characteristic parameters.

The AE characteristic parameters analyzed in this paper include ring count and AE energy, whose physical meanings are shown in Fig. 6. The cumulative ring count is the accumulated value of all AE event ring counts from the beginning of monitoring to a certain moment, reflecting the total damage occurring inside the material during fracture loading. A higher cumulative value indicates a greater total amount of microcracks induced within concrete, i.e., a higher degree of damage^38^. The cumulative AE energy is the sum of energies from all AE events, representing the total strain energy released due to microcrack initiation, propagation, and other forms of damage since loading began, directly reflecting the severity of material damage^39^.

**Fig. 7:**
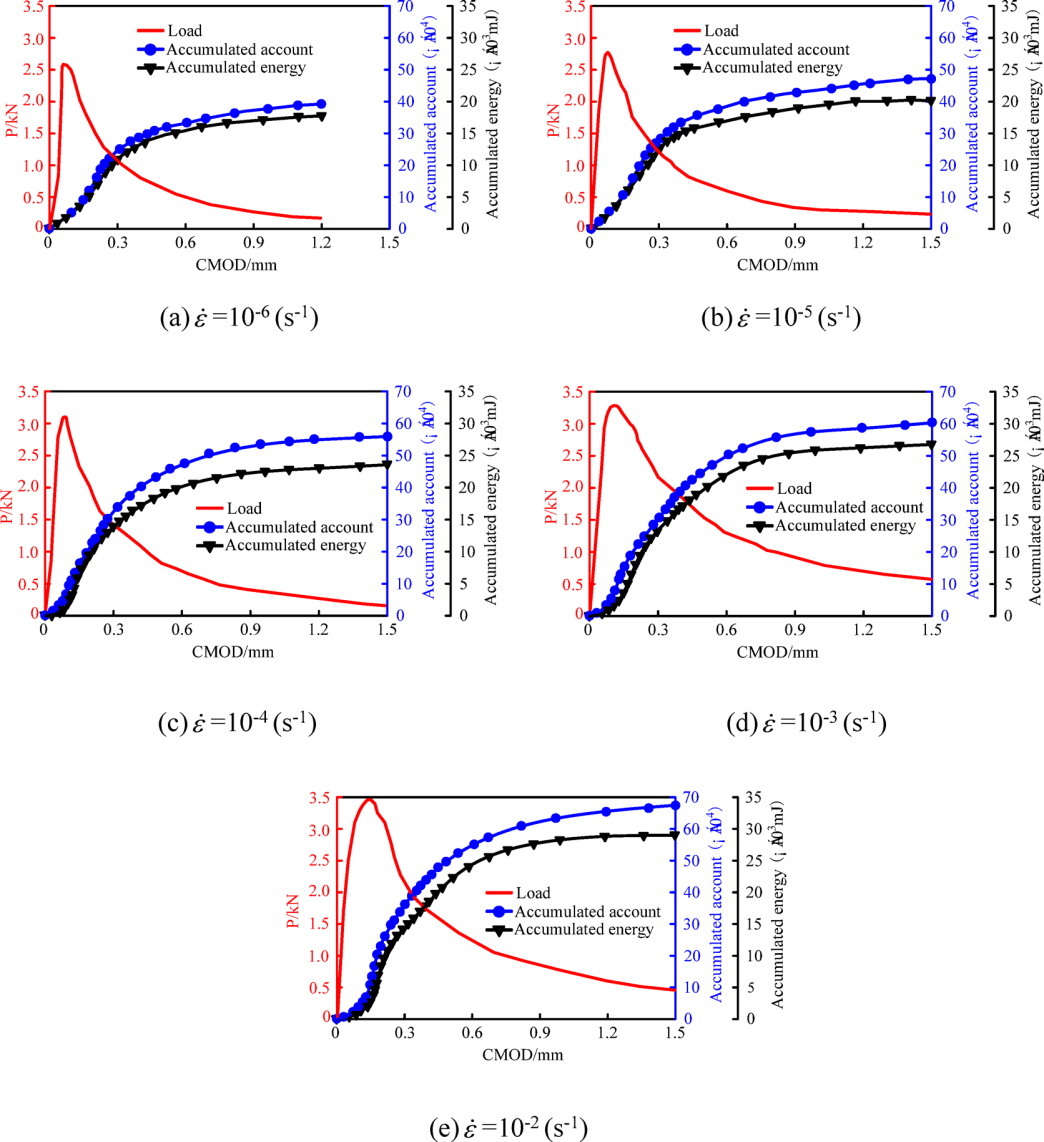
AE ringing count and cumulative energy of concrete under different strain rates.

Figure 7 shows the variation curves of concrete load, cumulative ring count, and AE energy with CMOD under different strain rates. As shown in the figure, both the cumulative ring count and cumulative AE energy exhibit a growth pattern characterized by slow increase—rapid increase—slow increase again, which corresponds to the three stages of crack initiation, stable propagation, and unstable failure in the P-CMOD curve. The cumulative ring count increases with increasing strain rate. When the strain rate increases from 10^− 6^ s^− 1^ to 10^− 5^ s^− 1^, 10^− 4^ s^− 1^, 10^− 3^ s^− 1^, and 10^− 2^ s^− 1^, the cumulative ring count increases by 20.1%, 42.5%, 52.6%, and 69.8%, respectively. This is because each initiation and propagation of a microcrack triggers an AE event. At low strain rates, crack paths mainly propagate along interfaces rich in pre-existing cracks or micro-pores; however, as the strain rate increases, crack paths become more direct, no longer propagating along ITZs but instead directly penetrating denser matrix materials or even stronger coarse aggregates. This means that under high strain rates, the number of newly formed cracks (i.e., AE sources) significantly increases, which is the main reason for the increased cumulative ring count. Similarly, the cumulative AE energy also increases with increasing strain rate. When the strain rate increases from 10^− 6^ s^− 1^ to 10^− 5^ s^− 1^, 10^− 4^ s^− 1^, 10^− 3^ s^− 1^, and 10^− 2^ s^− 1^, the cumulative AE energy increases by 11.1%, 33.4%, 49.8%, and 61.2%, respectively. The reason is simple: higher strain rates induce more microcrack initiations, meaning more channels for AE energy dissipation. Moreover, compared to interfacial debonding under low strain rates, the fracture of coarse aggregates at higher strain rates require more energy, which is primarily released in the form of AE energy and thermal energy.

To further understand the energy distribution during the fracture process, the relationship between Cumulative AE energy and *G*_F_ was analyzed. From the data in Tables 3 and 4; Fig. 7, it is evident that both *G*_F_ and AE energy increase significantly with the strain rate. For instance, when the strain rate increases from 10^− 6^ s^− 1^ to 10^− 2^ s^− 1^, *G*_F_ increases by 36.7% (from 91.7 to 125.3 N·m^− 1^), while the Cumulative AE energy increases by 61.2%. This synchronous growth suggests that the total energy dissipation within the material increases substantially under higher strain rates. Although AE energy constitutes only a small fraction of the total input energy (the majority being *G*_F_ and thermal energy), the consistent trend confirms that the intensification of micro-damage events (captured by AE) directly correlates with the macroscopic increase in fracture toughness.

### Location of AE-events and peak amplitude

By recording the propagating acoustic signals through various sensors and performing appropriate analysis to determine the source of these acoustic waves, this process is commonly referred to as acoustic source localization technology. When using the AE non-destructive testing technique for health detection and monitoring of concrete structures, accurately localizing AE sources is one of the primary tasks of this technology.

The time-difference location method to locate AE events was adopted. The parameters that need to be determined for the time-difference location method include (illustrated in Fig. 2 for the coordinate system): the propagation velocity *v*_*p*_ of the longitudinal wave in the concrete material, the distribution positions (*x*_*i*_, *y*_*i*_, *z*_*i*_) of each AE sensor, and the arrival times *t*_*i*_ of each AE sensor. Substitute all parameters into the Levenberg-Marquardt (LM) algorithm to inversely calculate the coordinates of the AE event. For the AE waveform signals of the same AE event, the velocity equation and the distance equation are shown as follows:

8 $${r_i}={v_p}\left( {{t_i} - {t_0}} \right)+\varepsilon$$

9 $${r_i}=\sqrt {{{\left( {{x_i} - x} \right)}^2}+{{\left( {{y_i} - y} \right)}^2}+{{\left( {{z_i} - z} \right)}^2}}$$

where *r*_*i*_ is the distance from the position of the AE event to the *i*-th AE sensor, *t*_0_ is the occurrence time of the AE event, (*x*, *y*, *z*) are the coordinates of the AE event, and *η* is the calculation residual. The formula for the sum of the squares of the calculation residuals is as follows:

10 $${\eta ^2}=\sum\limits_{{i=1}}^{n} {\left\{ {{{\left[ {\sqrt {{{\left( {{x_i} - x} \right)}^2}+{{\left( {{y_i} - y} \right)}^2}+{{\left( {{z_i} - z} \right)}^2}} - {v_p}\left( {{t_i} - {t_0}} \right)} \right]}^2}} \right\}}$$

where *n* is the number of AE sensors, *n* = 8. Using the idea of the least-squares method, optimize Eq. (3) step by step to obtain the minimum value of the sum of the squares of the calculation residuals *η*^2^, and then calculate the coordinates (*x*, *y*, *z*) of the AE event.

**Fig. 8:**
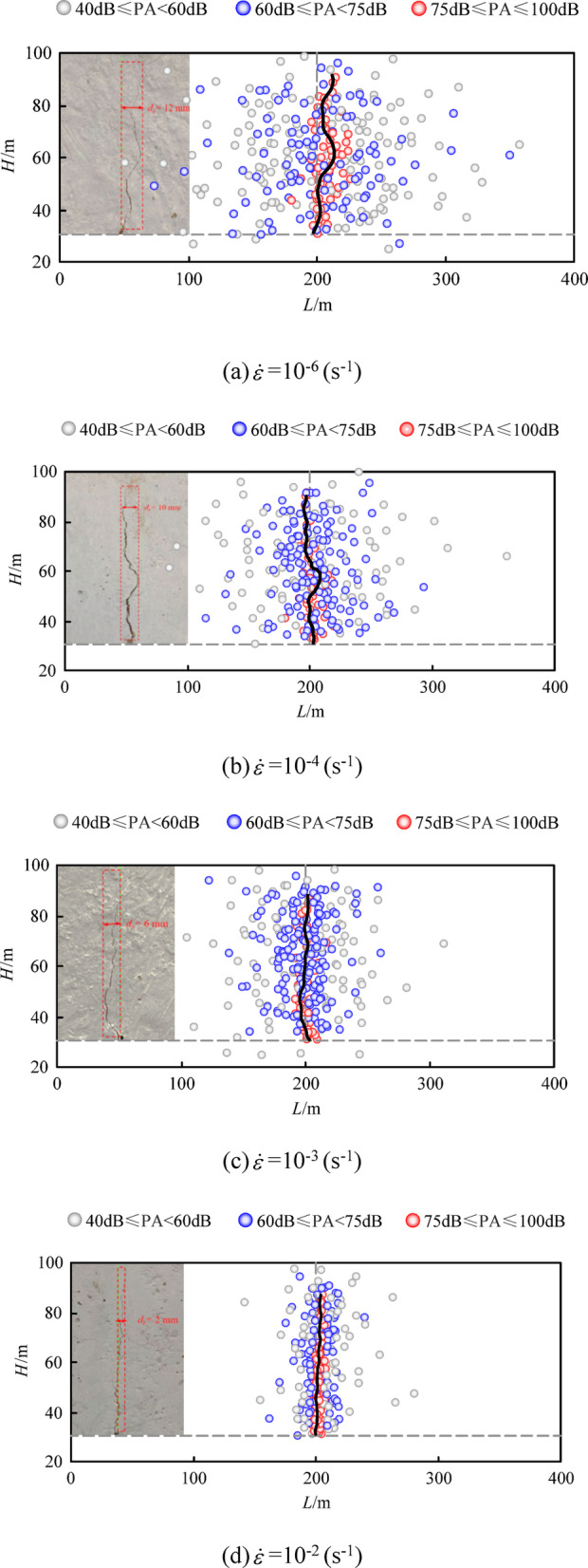
Spatial distribution of AE events in concrete fracture under different strain rates.

**Fig. 9:**
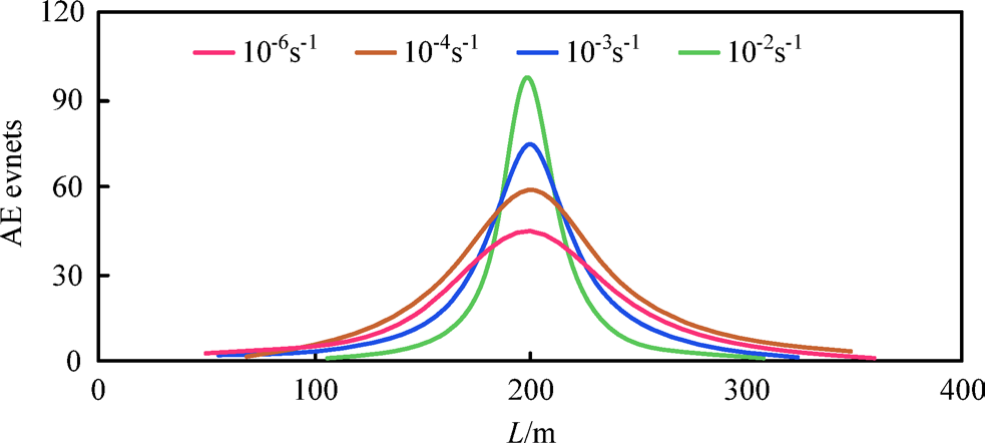
The distribution of AE event numbers along the long axis direction of the specimen.

To more effectively analyze the evolution behavior of concrete cracks, information on the corresponding peak amplitudes is supplemented based on understanding the distribution of AE events, as the peak amplitude of AE events can intuitively reflect the degree of material damage at different stages. The magnitude of the peak amplitude is determined based on the frequency of all AE events occurring in the specimen^40^. Concrete in a high-damage state releases more strain energy, which is then converted into high peak amplitude acoustic waves. In this experiment, the degree of material damage is classified into three levels based on the peak amplitude: minor damage (40 ~ 60 dB), moderate damage (61 ~ 75 dB), and severe damage (76 ~ 100 dB). Figure 8 shows the distribution of AE events and the corresponding peak amplitudes in concrete specimens under different strain rates, compared with the surface crack morphology at specimen failure, where “PA” is the abbreviation for peak amplitude. Figure 9 illustrates the distribution of AE event numbers along the long axis direction of the specimen. As can be seen that, throughout the loading process, AE events are basically symmetrically distributed along the central pre-existing crack in the length direction of the specimen, with an overall distribution density that is sparse at both ends and dense in the middle, resembling a normal distribution pattern. Moderately to highly damaged areas are mainly concentrated near the pre-existing crack, while minor damage areas are more dispersed and more uniformly distributed along the length direction of the specimen. High peak amplitude AE signals indicate that microcracks are converging into macrocracks, and the occurrence of a large number of high peak amplitude AE signals may imply that the specimen is about to undergo fracture failure. When the strain rate is low, the distribution of AE events is relatively scattered along the length direction of the specimen. As the strain rate increases, the events gradually converge toward the central pre-existing crack, and the density of AE events also increases. The higher the peak amplitude, the more pronounced this convergence effect becomes.

Comparison with the crack morphology after specimen fracture failure shows that the propagation path of the surface main crack is relatively consistent with the distribution of AE events, especially after the crack develops into a macroscopic one. The distribution positions of high peak amplitude AE events are basically located along the propagation path of the main crack. It is evident that the distribution pattern of AE events is closely related to the propagation trend of cracks, particularly the locations where high peak amplitude AE events appear, which can reliably predict the evolution path of fracture cracks. Additionally, as the strain rate increases, the morphology of the main crack propagation path changes from tortuous to straight, and the crack width decreases. Under the strain rates of 10^− 6^s^− 1^, 10^− 4^s^− 1^, 10^− 3^s^− 1^, and 10^− 2^s^− 1^, the crack widths of specimen are 12 mm, 10 mm, 6 mm, and 2 mm, respectively, indicating that the damage is more concentrated and the degree of damage is more severe. This is also an intuitive manifestation of the tendency for specimens to undergo brittle failure subjected to the load of the higher strain rate.

### RA-AF Values

**Fig. 10:**
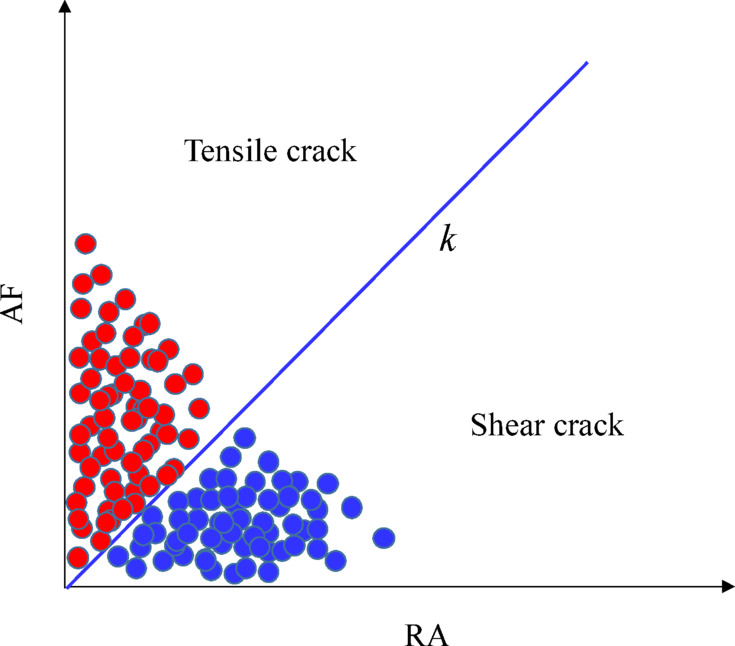
Diagram of RA-AF method for identifying crack types.

RA-AF (RA = Rise Time/Amplitude, AF = Ring Count/Duration, as illustrated in Fig. 6) combined analysis is a method based on the Japanese building standard JCMS-III B5706^41^. It is widely used for identifying the types of internal cracks in concrete, and its statistical analysis results can serve as indicators for quantitatively judging macroscopic fracture mechanisms. The ratio AF/RA is defined as *k*. When AF/RA < *k*, signals characterized by high RA and low AF are defined as shear cracks, while signals with low RA and high AF correspond to tensile cracks, as illustrated in Fig. 10. According to recommendations by Li^42^ and Ohno^43^, *k* value of 80 is considered appropriate.

To justify *k* value in our dynamic loading scenario, a cross-validation was performed by comparing the RA-AF classification results with the actual fracture surface morphology observed in Fig. 3. A strong correlation is found: at high strain rates where the *k* = 80 threshold predicted a higher proportion of shear cracks, the physical fracture surfaces indeed showed more transgranular (shear-like) features rather than interfacial debonding.

**Fig. 11:**
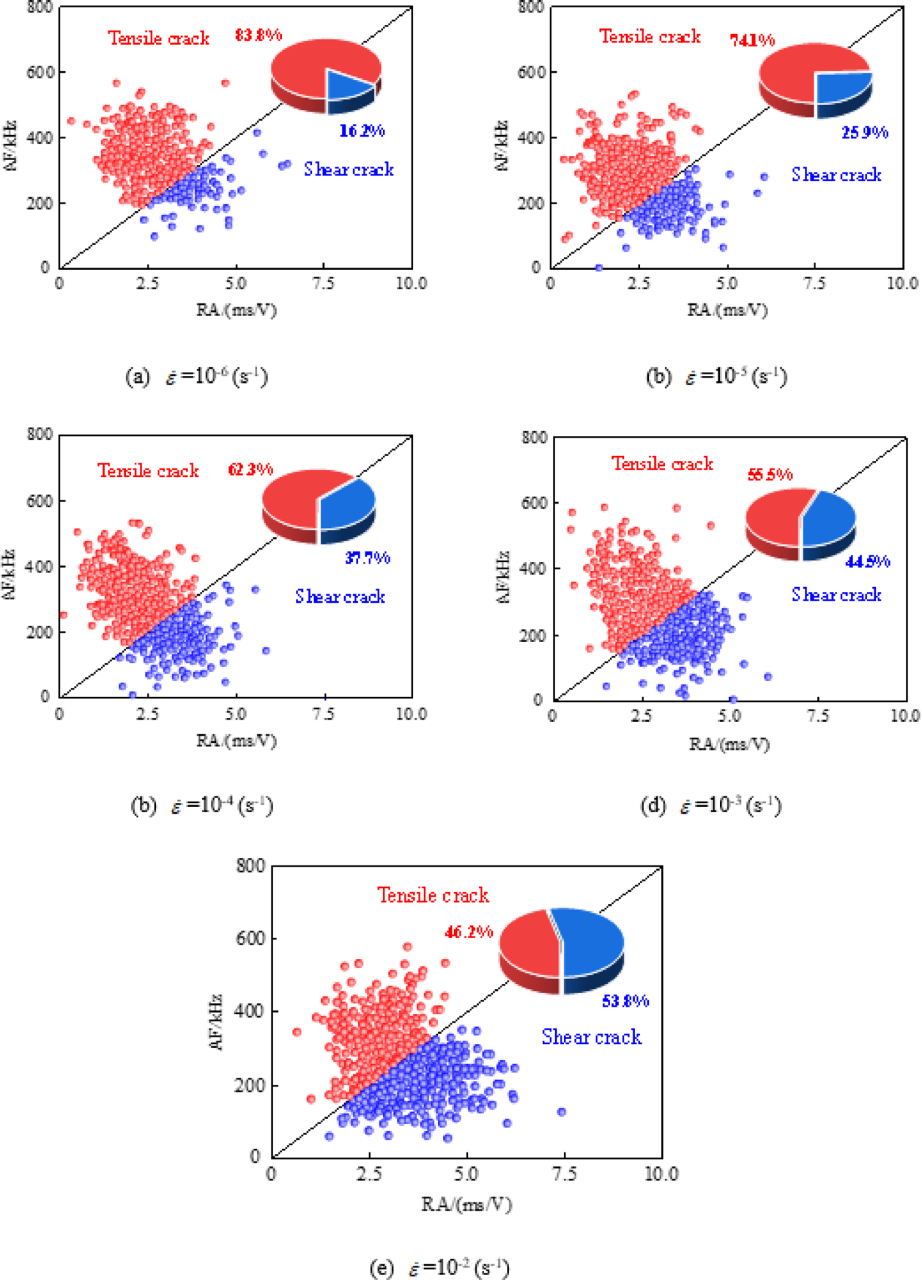
RA-AF distribution map of concrete under different strain rates.

Figure 11 illustrates the distribution of RA-AF values in concrete specimens under different strain rates, using a dividing line with *k* = 80 to classify crack types. The proportions and failure characteristics of different crack types are calculated. It can be observed that the RA and AF values of all specimen groups are mainly concentrated in the ranges of 2.5 ~ 5.0 ms·V⁻¹ and 200 ~ 500 kHz. Moreover, as the strain rate increases, the proportion of tensile cracks decreases while that of shear cracks increases. Taking shear cracks as an example, for specimens under strain rates of 10^− 6^s^− 1^, 10^− 5^s^− 1^,10^− 4^s^− 1^, 10^− 3^s^− 1^, and 10^− 2^s^− 1^, the proportions of shear cracks are 16.2%, 25.9%, 37.7%, 44.5%, and 53.8%, respectively. Thus, as the strain rate increases, the crack type gradually shifts from tension-dominated Mode I fracture to shear-dominated Mode II fracture.

The underlying reason is as follows: Under quasi-static loading (strain rates < 10^− 5^s^− 1^), tensile micro-cracks have sufficient time to nucleate diffusely. They propagate and connect along weak interfaces, such as the ITZ or existing defect regions, ultimately forming a macroscopic opening-mode (Mode I) fracture surface. During this process, stress relaxation at the crack tip is sufficient, and energy is primarily dissipated in the form of surface energy^44^. As the strain rate increases, the propagation speed of stress waves at the crack tip approaches the timescale of internal relaxation within concrete, hindering stress relaxation^45^. This leads to a sharp rise in the instantaneous shear stress component, causing a progressive transition in the fracture mode from Mode I to Mode II. Concurrently, the density of crack branching decreases, and the length of the primary crack shortens. This results in the intensive and interlaced formation of cracks within a narrow banded zone over a short period^46^. Macroscopically, this manifests as splitting-induced shear failure, which aligns with the phenomenon of numerous aggregates being sheared off on fracture surfaces under high strain rates, as observed in experiments(Fig. 3).

## Discussion

**Fig. 12:**
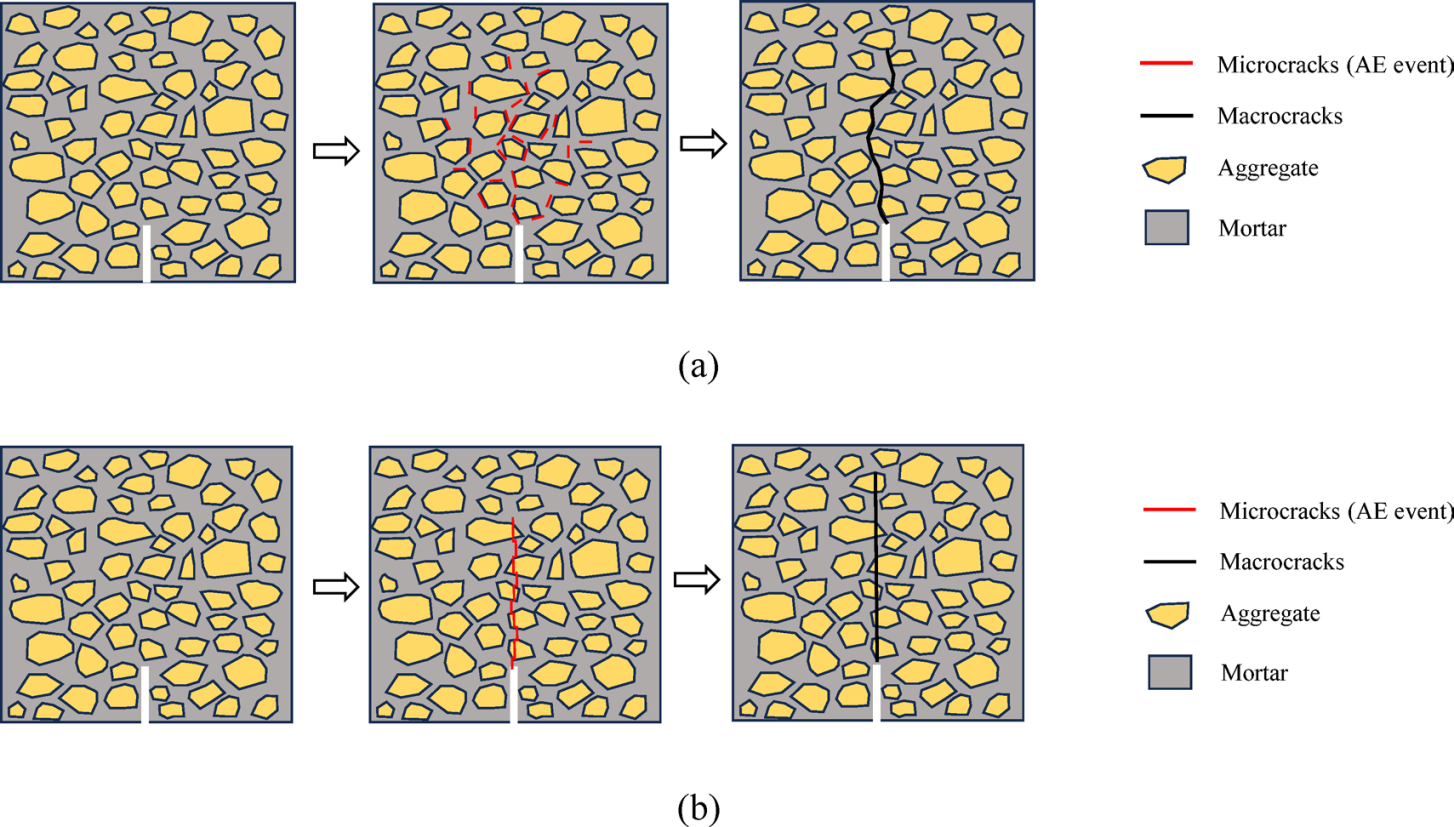
Microcrack and macrocrack formation process for concrete under different strain rates. (**a**) Lower strain rate, (**b**) Higher strain rate.

Within the strain rate range (10^− 6^ ~ 10^− 2^/s), concrete exhibits a pronounced strain-rate strengthening effect: increasing strain rate elevates fracture stress but promotes brittle failure modes. As illustrated in Fig. 12, under low strain rates (Fig. 12a), specimens have sufficient time to respond to localized stress concentrations, triggering widespread nucleation and slow propagation of microcracks (AE events) within the mortar matrix and at the ITZ, thereby forming relatively diffuse damage zones. The final macrocrack propagates along a tortuous path to circumvent rigid aggregates; although this mechanism dissipates considerable energy through extended crack paths and multiple microcracking—resulting in enhanced ductility^5–7^—it concurrently limits peak stress capacity. Conversely, under high strain rates (Fig. 12b), rapid loading induces swift stress wave propagation, preventing local stress relaxation. This leads to a sharp reduction in microcrack density and highly concentrated damage. Simultaneously, inertial effects impede crack deflection^4,17^, forcing cracks to propagate linearly along the shortest path—even directly fracturing aggregates. This straight-through fracture morphology significantly shortens the failure path while intensifying energy dissipation per unit time, enabling concrete to sustain higher stresses dynamically. Furthermore, as a strain-rate-dependent quasi-brittle material, the time-dependent damage mechanisms for the concrete—including subcritical crack growth and creep-induced degradation—are suppressed under high strain rates^17^. Consequently, the material reaches its extreme state before accumulating sufficient damage, further enhancing dynamic strength. However, this strength gain comes at the expense of ductility: concentrated crack development under high strain rates predisposes structures to sudden, catastrophic failure, undermining their warning capacity. Specifically, the observed transition from a tortuous, Mode I-dominated fracture mechanism (characteristic of low strain rates) to a straight, Mode II-dominated shear fracture under rapid loading has critical implications for seismic design. In shear-critical structural members—such as beams, columns, or shear walls—this shift indicates a heightened risk of brittle shear failure over ductile flexural failure during seismic events. The high-strain-rate environment prevents the energy-dissipating mechanisms associated with microcrack branching (Mode I) and forces cracks to propagate linearly (Mode II), significantly reducing the member’s toughness. Therefore, in seismic engineering design, it is essential not only to leverage strain-rate-induced strength enhancements to avoid overly conservative estimates but also to remain vigilant against the associated risk of brittle failure. Engineers must account for the strain-rate dependency of the fracture mode when assessing the shear capacity of critical members. Strategies such as optimized reinforcement detailing or incorporation of toughening agents (e.g., steel fibers) should be employed to improve dynamic fracture toughness.

## Conclusion

(1) Concrete exhibits significant rate-dependent pseudo-strengthening. With the strain rate from 10^− 6^s^− 1^ to 10^− 2^s^− 1^, the peak load (*P*_max_) increases by 37.1%, rising from 2.59 kN to 3.55 kN, and fracture energy (*G*_p_) increases by 36.7%, from 91.7 to 125.3 N·m^− 1^, while the unstable toughness double from 0.60 to 1.23 MPa·m^1/2^.

(2) At high strain rates, the crack path transitions from deflection along the ITZ to direct penetration through coarse aggregates (transgranular cracking). This forced penetration of rigid aggregates consumes substantial energy, explaining the elevated *G*_p_ despite the loss of ductility.

(3) The failure mechanism shifts from Mode I (tensile) to Mode II (shear) dominance, with the strain rates increases. RA-AF analysis shows the proportion of shear cracks increases from 16.2% to 53.8%. Rapid loading prevents stress relaxation, causing high shear stress concentrations at the crack tip. Consequently, cracks propagate linearly rather than deflecting, resulting in a mixed-mode fracture that is increasingly shear-controlled.

(4) AE source distribution reveals a transition from diffuse damage to localized concentration. High strain rates restrict microcrack development to a narrow zone near the pre-crack tip. While this concentration allows higher stress bearing, it eliminates the warning signs of failure. This indicates that dynamic loading transforms concrete into a brittle material with reduced ductility and energy redistribution capacity.

(5) The findings underscore the necessity of incorporating strain-rate sensitivity into seismic design codes and structural health monitoring systems. As concrete loses ductility and transitions to brittle failure under rapid loading, traditional static design assumptions may underestimate the risk of sudden collapse. Engineers should prioritize enhancing shear resistance and develop real-time monitoring strategies that detect the shift from tensile to shear-dominated cracking to ensure the resilience of concrete structures during seismic events.

## Data Availability

The authors confirm that the data supporting the findings of this study are available within the article [and/or] its supplementary materials.
